# Mechanical Pull-Out Test of a New Hybrid Fixture-Abutment Connection: An In Vitro Study

**DOI:** 10.3390/ma14061555

**Published:** 2021-03-22

**Authors:** Gianmaria D’Addazio, Bruna Sinjari, Lorenzo Arcuri, Beatrice Femminella, Giovanna Murmura, Manlio Santilli, Sergio Caputi

**Affiliations:** 1 Department of Innovative Technologies in Medicine and Dentistry, University “G. d’Annunzio” of Chieti-Pescara, 66100 Chieti, Italy; gianmariad@gmail.com (G.D.); beatrice.femminella@yahoo.it (B.F.); giovanna.murmura@unich.it (G.M.); santilliman@gmail.com (M.S.); scaputi@unich.it (S.C.); 2 Department of Oral and Maxillo-Facial Sciences, “Sapienza”, University of Rome, 00185 Rome, Italy; lorenzo.arcuri.1990@gmail.com

**Keywords:** implant-abutment connection, pull-out test, dental implant, implant success, conical connection, hybrid connection, microgaps

## Abstract

Implant abutment connection was described among the main causes of peri-implant bone resorption. The aim of this in vitro study was to test the coupling capacity, the surface modification of a new hybrid connection and the influence of repeated connection activations caused during the main clinical and laboratory phases. A total of 40 implant-abutment screw retained systems with 10°-conical and internal hexagon connection were tested. The connection was screwed, fixed to the universal test machine, removed the screw and a pull-out test was performed. Test was repeated five times in succession. Also Scanning Electron Microscopy (SEM) was used to detect microscopically surface modification. Analysis of variance and Tukey tests were used for the statistical analysis. Pull-out test reveals a mean value of 131.35 ± 16.52 Newton Centimeter (N·cm). For each single activation, results from first to fifth were: 113.9 ± 13.02, 126.1 ± 12.81, 138.11 ± 15.15, 138.8 ± 11.90 and 140 ± 12.99 N·cm. A statistically significant difference between the measurements and an increase in the removal force was shown. The collected data supports the use of this new type of connection, resulting in a very strong interface between implant and abutment. Also, repeated activation of connection can promote a better coupling of the implant-abutment interface.

## 1. Introduction

Peri-implant crestal bone resorption has been described as one of the major complications associated with maintenance and success in implant therapy [1,2,3]. Various factors have been described as responsible for crestal bone resorption, including biological width formation, surgical trauma and prosthetic complication like overload and fracture [4,5,6,7]. Nevertheless, the fixture abutment connection remains also a key factor [2]. It has been described how many elements could affect this critical area, such as fixture and abutment material, surface microtopography, treatments and connection type [8,9,10]. The latter could influence the biological response of peri-implant hard and soft tissues. Connection type and its stability are therefore directly related to the inflammatory response generated in the peri-implant soft tissues [1]. In fact, bacterial load within the connections due to the presence of a microgap could influence peri-implant microenvironment [11]. The microgap has been described as the microscopic space between the implant and the abutment once these two pieces are connected together. The goal of implantology nowadays is to avoid the presence of the microgap on two pieces implants to reduce the bacterial infiltration, inflammatory response and micromovements present at the interface [12].

Regarding these, different solutions of implant-abutment connections have been studied [13], discussing the advantages and disadvantages of each type. The first type of implant-abutment connection described was the external hexagonal connection [8,14]. It presented an easy prosthetic system to be used for placement and removal of abutment, making the everyday prosthodontics more friendly. Moreover, the external hexagon allows the management of moderate disparallelisms. On the other hand, the external hexagon had the limitation of a portion of anchorage very reduced in height, with approximately 0.7 mm. This problem made the connection brittle, especially in relation to lateral load forces [15]. In order to reduce these problems, the internal hexagon design provided greater resistance to side loads and better forces distribution [13]. However, the constant presence of a microgap at the interface still drives the search for an alternative solution [8,13,16]. In particular, Assenza et al. in 2012 demonstrated how even a cemented connection can eliminate the presence of the microgap [16]. The disadvantage of the cemented connection type is the probability of residual cement on the peri-implant soft tissues leading to inflammation or infection [17]. Furthermore, conical connections have been shown to have better microgap sealing capability than other connections, effectively reducing bacterial colonization thus reduce inflammation [18]. Additionally, conometric connections contribute significantly to the reduction of micromovements [18], but the lack of anti-rotational system could generate a rotation of abutment and could be sometimes hard to use in the dental practice [19]. However, recently, hybrid connections have been used in order to match conical and internal hex connection advantages: sealing capacities of the conometric connections with the easy-to-use of internal hexagonal connections in order to improve the clinical practice and implant long term success rate [20]. 

Many studies over the years have verified the capabilities of the above-mentioned types of connections in terms of mechanical characteristics after loading [21,22]. Some of them have shown how a plastic deformation occurs clearly at the abutment implant interface after use. Moreover, the cyclic load has been widely used to better analyze the mechanical behavior overtime, by demonstrating that the type of material and load direction could affect the deformation and micro-movements at the interface [21]. Some authors have shown that even early alterations can occur in the abutment implant system [2,4,8,22]. For this reason, it is important to check the sealing capacity of the system and the possible influence of the early clinical work phases. The aim of this experimental in vitro study was to verify the coupling capacity, the microscopically surface modifications of a new hybrid connection and the influence of repeated connection activations. The null hypothesis is that there are no differences following the activation of the new implant abutment connection.

## 2. Materials and Methods

### 2.1. Implant-Abutment Systems Characteristics and Study Design

**Figure 1:**
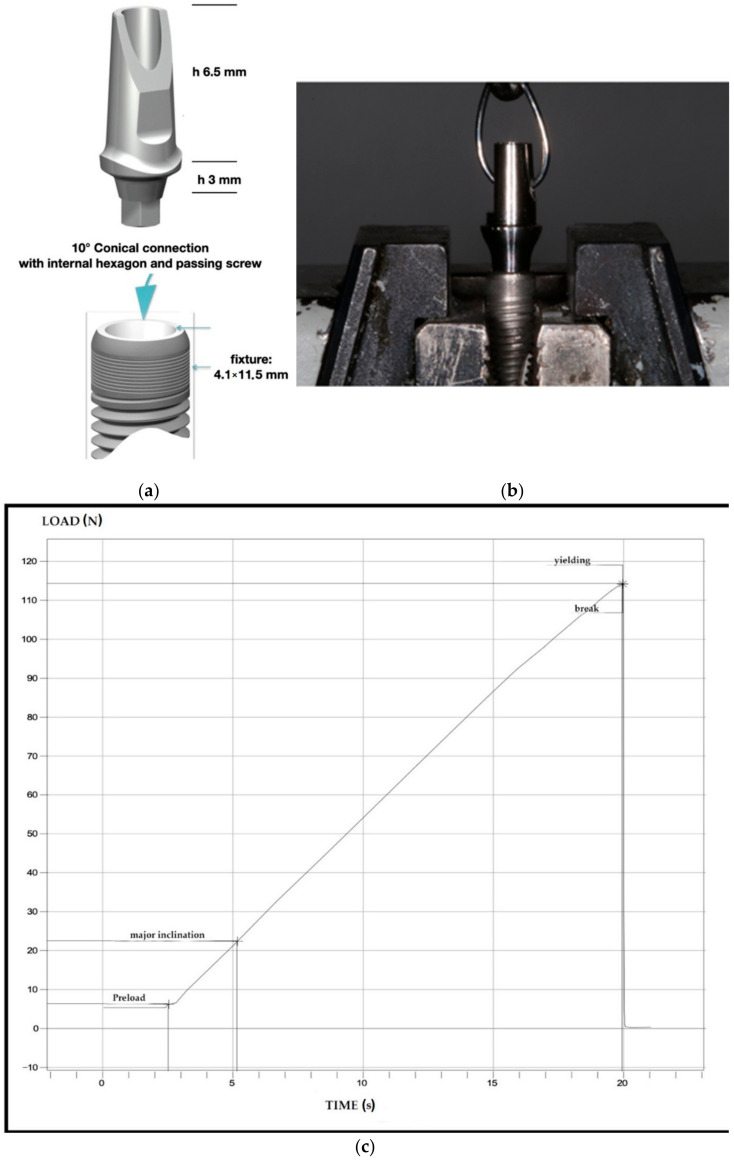
(**a**) Technical specific dimensions. (**b**) The fixture-abutment system fixed to the universal test machine. (**c**) Graphical representation of load-time curve.

A total of 40 commercial dental implants (LEADER Italia s.r.l., Cinisello Balsamo, Italy) with 4.1 mm diameter and 11.5 mm length and their relative abutments were used in this in vitro study. A new hybrid conical connection called morse connection (MC) was used, composed by an inner hexagon to locate the abutment, a 10-degree conometric connection with a screw useful to activate the latter and a transgingival potion of 3 mm. The mechanical tests were performed at the laboratory of the Department of Innovative Technologies in Medicine and Dentistry, University “G. d’Annunzio” of Chieti-Pescara. The specific dimensions are reported in Figure 1a. 

### 2.2. Mechanical Test

Once the abutments were coupled with specific dental implants, the connections were screwed at 25 Newton centimeter (N·cm) as suggested by the manufacture. Furthermore, after 2 min a new tightening at the same torque was performed to reduce the settling effect, as reported previously [23]. As shown in Figure 1b, the fixture-abutment system was fixed to the universal test machine (Lloyd LR30K, Lloyd instruments; Fareham, UK) perfectly parallel to perform the loading tests. Fixtures were blocked at the base of the test machine and the abutments drilled, whether the abutments lose the connection was controlled, and then the screws were removed. The drilled abutments were attached with a chain to the hook and tensioned. A preload was set to 6 N·cm to avoid system shifts. The abutment removal test was achieved (pull-out test) in order to evaluate seal and cone-coupling validity. The test machine was set to pull out until the abutments decoupling with a controlled and continuous force was achieved. The test machine speed was equal during the whole test. A graphical representation was obtained after each test, resulting in a loading-time curve as shown in Figure 1c. The same test was repeated five times in succession for each abutment screwed, by paying attention to replace the abutment at the same identical position. The hole in the abutment helped to perform this and locate the abutment. 

### 2.3. Scanning Electron Microscope (SEM) Test

Implant abutment connections were observed using an SEM (EVO 50 XVP with LaB6; Carl Zeiss S, Oberkochen, Germany), according to a previously described procedure [24,25]. 

Samples were metallized by a gold-sputter Emitech K550 (Emitech Ltd., Ashford, UK) and subsequently inserted into the sample-holder for SEM analysis. The SEM was equipped with a tetra solid-state back-scattered electron detector, set to operate at 30 kV accelerating voltage, 10 mm working distance and 870 pA probes current. The images were captured with 20 scans using a line-average technique. The obtained images were then analyzed using ImageJ software 1.48f 3D (Wayne Rasband, National Institutes of Health NIH, Bethesda, MD, USA), in order to obtain information about the microscopic modification in the implant-abutment interface area before and after the mechanical pull-out tests.

### 2.4. Statistical Analysis 

Mechanical tests data were collected and then used for statistical analysis. Specifically, the results are presented as mean and standard deviation. One-way analysis of variance (ANOVA) and Tukey tests were used to evaluate the overall significance and to perform all pairwise comparisons of the measurements at the different time points activation. A *p*-value of <0.05 was considered statistically significant. Statistical analyses were performed using the GraphPad version 8 (GraphPad Software 2365 Northsides Dr. Suite 560, San Diego, CA, USA) statistical software.

## 3. Results

**Figure 2:**
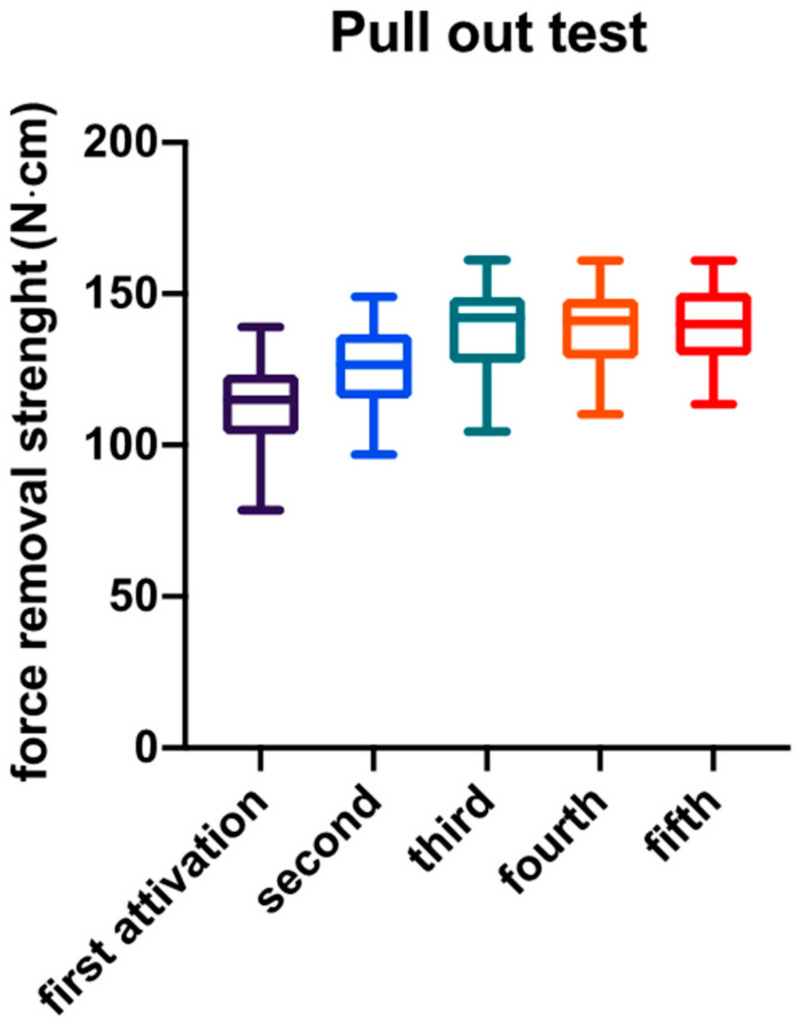
Mean value and standard deviation of pull-out test at different time points.

All the abutments successfully completed the test. No fracture or detachment of the components were recorded. The force removal strength values and the graphical representation were recorded. The 40 samples were tested five times each and the average removal force between fixture and implant was 131.35 ± 16.52 N·cm. Furthermore, the 40 samples were measured at each single activation, obtaining the mean following results: 113.9 ± 13.02 N·cm at the first activation, 126.1 ± 12.81 N·cm at the second activation, 138.11 ± 15.15 N·cm at the third activation, 138.8 ± 11.90 N·cm at the fourth activation and 140 ± 12.99 N·cm at the fifth activation. Figure 2 shows the mean value of each single activation. Table 1 shows a descriptive statistic of the obtained results. The ANOVA test showed a statistically significant difference between the measurements (*p* < 0.0001). In addition, carrying out the Tukey multiple comparison test details of the comparison between the different activations demonstrates that there was a statistically significant difference between the first three activations. Meanwhile, an increase in the abutment removal force during the different time points was observed in all the specimens tested. On the contrary, no statistically significant difference was demonstrated by comparing activations from the third onwards. Multiple comparison analysis is reported in Table 2.

**Figure 3:**
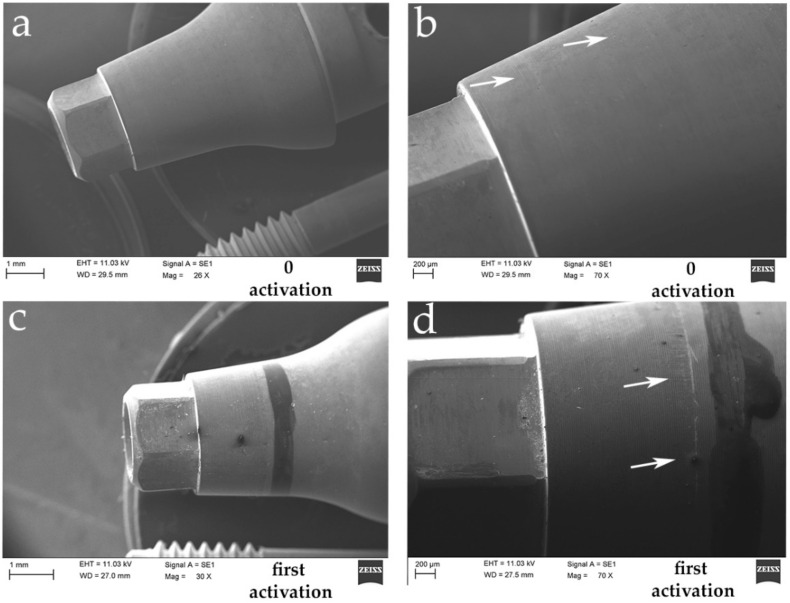
The SEM images show two different magnifications on the abutment surface: (**a**) 26× and (**b**) 70×, before activations. No apparent alteration of the titanium is detectable. A wavy pattern is visible as a result of the manufacturing process of the abutment itself. (**c**) 30× and (**d**) 70×. The arrows indicate the contact portions between the abutment and the fixture. Vertical streaks and irregularities can be interpreted as points of greatest friction in the connection.

**Figure 4:**
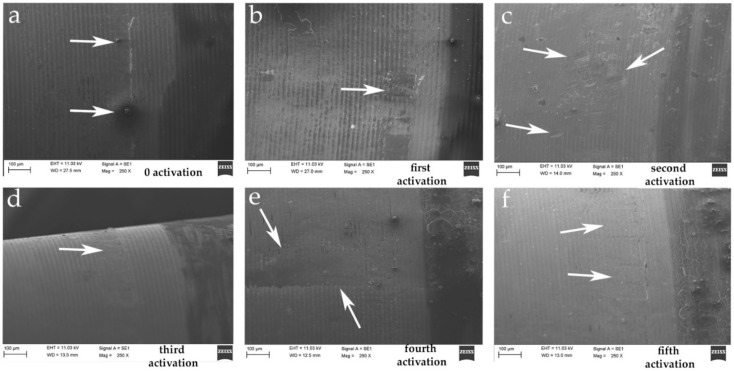
SEM images at higher magnification (250×). (**a**) No activation. Arrows show an irregularity into the interior part of abutment never activated. (**b–f**) From first to fifth activation, an increase in vertical streaks is visible on the abutments (white arrows). This can be interpreted as points of greater friction of the connection.

The SEM investigation showed a difference on the surface of the never activated abutments and on the abutments after multiple activations, where scratches and many sorts of fretting wear on the surface of the connection were shown. The microscopic image showed on the coupled abutments irregular shapes and grooves not present on the new abutments. Moreover, on the new abutments there were coiled furrows with a wavy pattern, as shown in Figure 3. On the other hand, irregularities and elongated and sharp grooves were present on the activated abutments as shown in Figure 3 and Figure 4. The precise points where the greatest friction of the system caused a dent on the abutment are clearly visible in Figure 4 at the different activation point. 

**Figure 5:**
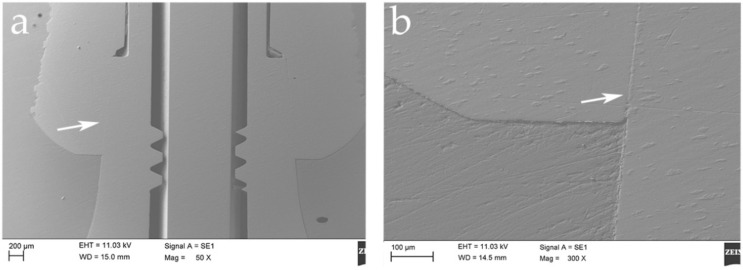
SEM images showed the microgap between implant and abutment, indicated by arrows. (**a**) 50× magnification and (**b**) 300× magnification.

As anticipated, the wavy horizontal streaks are also present on the abutment that has never been activated, thus demonstrating how they are an integral part of the processing of the abutment itself. Differently, the vertical streaks turn out to be scars from rubbing or scratches on the surface which result as microscopic changes caused by the activation of the connection itself. In Figure 4, it is possible to identify a greater number of streaks caused after repeated activation of the abutment itself. There seem to be random scratches that are always present in the same longitudinal activation zone of the abutment, as if they were major contact points of the abutment itself. This damage appears to increase significantly after repeated activation. All series of images were obtained on the same gray scale to compare the irregularities before and after activation and to be able to highlight the microgap at the interface between implant and abutment. In Figure 5, the microgap is shown at the interface at the first and at the fifth activation, demonstrating a very reduced space with this kind of connection. 

## 4. Discussion

The null hypothesis under test was rejected. The results showed a statistically significant difference in terms of removal torque force in the samples during the different activation (*p* < 0.0001). Tukey’s test revealed that there are differences between groups but not between all activation time points. Compared with others type, these results seemed very interesting [26,27]. In fact, Hsu et al. in 2018 showed in implants with conical connection a pull-out test value of 77.60 N·cm. The same authors measured this value before the load tests obtaining 55.28 N·cm [26], confirming our trend. Specifically, a statistically significant increase in removal force was demonstrated during the first three activations in our study. Moreover, from the third activation onwards, a further increase in activation force was demonstrated but this was not statistically significant (*p* > 0.05).

Additionally, Pintinha et al. in 2013 obtained values of 50.96 N·cm, meaning again an increased value, after loading tests [27]. The authors explained that an increased pull-out force could result from the type of angle used in the connections [26,27,28]. In the past, other authors stated that implants with conical connection having a smaller tapered angle could lead to a greater pull-out force, due to a higher axial displacement [26,29,30]. Clinically, this translates into a reduction in micro-movement and a lower possibility of removal of the abutment [26,30].

In addition to the angle, there are other factors that can influence the pull-out test. These include surface area of connections between implant and abutment, tightening torque values, friction coefficient, etc. [31,32]. Furthermore, the settling effect could be an important aspect to observe in order to guarantee the integrity of the coupling system [23,33]. The settling effect is generated since neither the interior part of implant-abutment system nor the screw is perfectly fabricated and without irregularity [23]. Regarding the screw, a strict protocol was followed during experiments to reduce the possibility of generating the settling effect. In particular, the tightness of the screws is ensured by the preload generated during tightening. It has been demonstrated that a correct screw tightening and a further second tightening after 2 min, guarantee a statistically significant reduction in the loss of force after loading [23].

At the same time, the above-mentioned results and SEM images have shown that repeated activations (for example during the clinical and laboratory phases) can reduce potential friction in specific points of the abutments. Moreover, the repeated activations can increase the extension of the connection area itself, demonstrated by an increase in the pull-out force of the abutment. The irregularities themselves can be interpreted as points of greater friction present before the activation of the abutment, which appear more regular after the activation of the connection itself. The factors described must be kept under control to reduce the possible micro-movements generated by the union of the two components (implant and abutment). Micro-movements between the abutment and the internal part of the implant can increase the stress at the marginal bone level [34]. At the same time, it can change the volume of the internal space of the implant-abutment complex, causing a resulting pumping effect that can also carry microorganisms from the outside to the inside and vice versa [18,35]. This probably may have repercussions on the maintenance of the peri-implant bone during time [36]. Many authors have demonstrated the presence of microgap and micro-movements of the system among the causes of peri-implant bone resorption [2,8,22]. Mechanical aspects are also closely related to the stress-shielding phenomena. In this sense, the overload and therefore the presence of micro-movement can negatively affect the mechanical forces distribution to the surrounding bone, causing its resorption [37]. Several authors have emphasized how connections subject to micromovements under load can create a microgap at the abutment–fixture interface [35,36,38,39]. Our SEM images showed the presence of an extremely small microgap. Other authors used pure conometric connections demonstrating that it eliminates the microgap [18,39]. On the contrary, other authors have shown that even in conometric connections, a total elimination of the microgap is not possible [38]. Instead, cemented connection has shown total sealing of microgap [16,40]. Assenza et al. in 2012 demonstrated a perfect seal without microbiological leakage [16]. Subsequently, Sinjari et al. in 2019 have shown how even after 10 years of function, this type of connection presented a reduced marginal bone loss compared to internal hexagon, with an extremely low failure rate (in terms of debonding) [40]. Otherwise, the possibility of having a conometric connection with a screw and an anti-rotational system makes clinical practices easier [38,41].

Our goal was to test the reliability of abutments made by the manufacturer, on a single implant platform to verify the effectiveness of the implant production process. The majority of the authors who test axial displacement use cyclic or static loads tests. Furthermore, they use customized abutments, which can affect results. As demonstrated in this in vitro study, this can be influenced already from the early stages of function [26]. It has already been shown how the application of compressive force can increase frictional resistance resulting from the contact of the conical portions [42]. Studies carried out with finite element (FE) models have shown that more than 86% of the tightening torque and over 98% of the relaxation torque are balanced by the conical joint of these systems [42]. Generally, most studies dealing with axial displacement do this during cyclic loads [30,43] and few studies have dealt with static loading. Ko et al. [44] reported that axial displacement and removal force dramatically decrease as a result of cyclic loading. Furthermore, the very common use of customized abutments in dentistry made with computer-aided design and manufacturing (CAD/CAM), showed differences during axial loads, probably related to the different production techniques of the abutments themselves. Contrarily, the abutments made by the manufacturers present a higher breaking strength (>700 N) being only affected by the different types of connection used. Despite the extreme precision of the MC connections observed, in the present study, the irregularities on the surface may generate friction which reduces the seal of the abutment itself. Multiple activation, in the early stages of work, can significantly improve the mechanical performance of the abutment.

Being an in vitro study, this clearly has some methodological limitations. The goal is to use these components for future clinical studies so as to validate their long-term efficacy and any influences on peri-implant bone resorption. No cyclic loading was performed. Also, the analysis of non-axial loads on the same systems may be a subject of study. SEM images clearly showed the alterations on the inactivated and repeated activated abutments. Other analysis, such as the depth of traces or the distance between traces, could be investigated. Finally, a larger number of samples could allow expansion of the most interesting aspects of the current research.

## 5. Conclusions

The pull-out values of the abutment expressed in N·cm were particularly encouraging. The collected data supports the possibility of clinical study or application and also dynamic mechanical testing. The possibility of merging the clinical simplicity of the internal hexagon and the safety of conical coupling supported the use. The collected data showed the possibility of reducing abutment micromovements due to the high removal force. Moreover, a very reduced microgap was demonstrated. The repeated activation of the connection in the early stages of work can increase the performance of the connection itself.

## Figures and Tables

**Table 1:** Descriptive statistics of pullout test.

Descriptive Statistic	Abutment Activation				
	First	Second	Third	Fourth	Fifth
Measurements (n)	40	40	40	40	40
Minimum	78.54	96.87	104.4	110.2	113.5
25% Percentile	103.5	115.4	127.2	128.6	129.4
Median	114.9	126.6	142.0	141.1	140.0
75% Percentile	123.4	136.8	148.8	148.3	150.4
Maximum	139.1	149.1	161.2	161.0	161.0
Mean	113.9	126.1	138.1	138.8	140.0
Std. Deviation	13.02	12.81	15.15	11.90	12.99
Std. Error of Mean	2.059	2.025	2.396	1.881	2.053
Lower 95% CI	109.7	122.0	133.3	135.0	135.8
Upper 95% CI	118.0	130.2	143.0	142.6	144.1

**Table 2:** Post-hoc Tukey’s multiple comparison tests performed for investigate change between different activation time point. Level of significance: ns (non-significant), *** (*p* < 0.001), **** (*p* < 0.0001).

Comparison	Tukey’s Multiple Comparisons Test			
	Mean Difference	95% Confidence Interval of Difference	Significance	Level of Significance
1° vs. 2°	−12.20	−20.33 to −4.058	0.0005	***
1° vs. 3°	−24.23	−32.37 to −16.10	<0.0001	****
1° vs. 4°	−24.89	−33.02 to −16.75	<0.0001	****
1° vs. 5°	−26.08	−34.21 to −17.94	<0.0001	****
2° vs. 3°	−12.04	−20.18 to −3.901	0.0006	***
2° vs. 4°	−12.69	−20.83 to −4.554	0.0003	***
2° vs. 5°	−13.88	−22.02 to −5.745	<0.0001	****
3° vs. 4°	−0.6530	−8.790 to 7.484	0.9995	ns
3° vs. 5°	−1.844	−9.981 to 6.293	0.9711	ns
4° vs. 5°	−1.191	−9.328 to 6.946	0.9944	ns

## Data Availability

The data presented in this study are openly available in Zenodo at 10.5281/zenodo.4625437.
